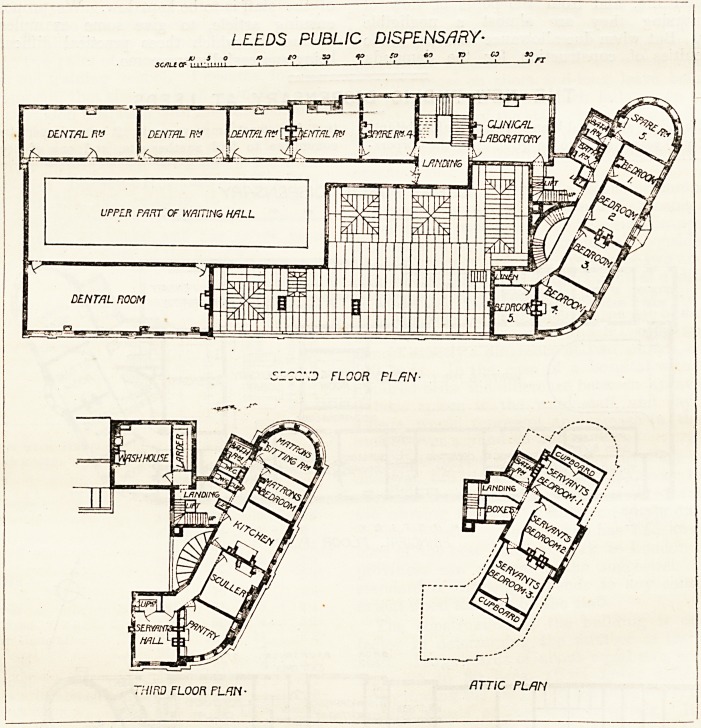# The New Public Dispensary at Leeds

**Published:** 1905-08-26

**Authors:** 


### THE NEW PUBLIC DISPENSARY AT LEEDS.

THE old building which had been known to the inhabitants of Leeds for 40 years having to be given up to the Corporation for street improvements, it was imperative on the committee to find another site, and one of nearly 1,500

square yards was obtained in Hartley Hill, having also frontages in Back Brunswick Street and in North Street. A limited number of architects was invited to compete for plans for the new work, and the plan finally selected was that by Messrs. Bedford and Kitson.

Advantage has been taken of the difference in level of the various portions of the site. The main entrance faces North Street and gives access to the board-room, the secretary's office, and the residential portion of the dispensary, while the upper door in Hartley Hill admits to the out-patients department, and the lower door in the same street forms the entrance to the casualty department. There is no doubt about the general advantage of this arrangement, as it will

On the first floor front in North Street, which is on a level with the out-patients' entrance at the upper part of the Hartley Hill front, are the medical officers' rooms; and along Hartley Hill are the surgeons' consulting-rooms and examining-rooms, sterilising-room, operating theatre and ophthalmic-rooms, all very cleverly and conveniently arranged.

The central portion of the dispensary contains a fine waiting-hall, measuring 90 feet long by 30 feet wide. Next to it is the nurses' room, and then dispensary lobby and dispensary. On the Back Brunswick Street front are the main staircase, physicians' consulting-rooms and examining-rooms, lavatories, and isolation-room.

prevent any likelihood of clashing between the various departments. The residential portion is contained in this front block facing North Street. On the right of the entrance is the board-room, and on the left are the secretary's office and the faculty sitting-room; and behind these is the staircase for the resident officers. A back staircase with lifts attached is approached from the back part of the hall beyond the second vestibule door.

The casualty entrance is on the lower part of Hartley Hill. This department has two casualty-rooms, waiting-room, staircase to dispensary, hall porter's room, sitting-room, and bedrooms. In Back Brunswick Street are the dispenser's rooms, and there is a general exit from this floor on to the street.

On the second floor are the bedrooms for the resident medical officers; and, passing along to the Brunswick Street front, we find the clinical laboratory, the staircase, and four dental-rooms. On the Hartley Hill front of this floor is another large dental-room. All these rooms are reached from the waiting-hall by means of a staircase to a gallery, which runs completely round the upper part of the waiting-hall.

The matron's rooms are on the third floor, and so are the kitchen and the washhouse; while the domestic servants are accommodated on the attic floor.

On every floor and on every aspect there is abundance of light. It will be seen from this description, and from the plans which we publish herewith, that an immense amount of accommodation has been obtained on a site which does not quite reach 1,500 square yards; and we think that the dispensary committee and the architects are much to be congratulated on the result of their work. We wish we could have given some particulars as to the total cost of the building, which we believe was formally opened this year (1904) by Dr. Clifford Allbutt, of Cambridge.

As to the details of construction we notice that the walls of the operating theatre are lined with tiles; those of the isolation-room are of glazed brick, and the other rooms are faced with granite plaster. The floors of the operating theatre, the casualty department, and the isolation-room are covered with marble terrazzo, and those of the waiting hall, dispensary-lobby, etc., are of maple-wood blocks. Hot-water radiators are used for warming, and electric fans for ventilation; but we are glad to notice that radiators are not exclusively relied on for heating, and that the "Teale" grate is in use.

Between the years 1867 and 1903, the old dispensary treated 1,052,463 patients at an average cost of 1s. 5¾d. per head. The new and larger dispensary will of course require a higher income for its adequate support; and it is not without good reason that the committee ask the people of Leeds to increase their subscriptions in aid of a charity by which so much suffering is relieved and which, unlike many other charities, is so little abused.

## Figures and Tables

**Figure f1:**
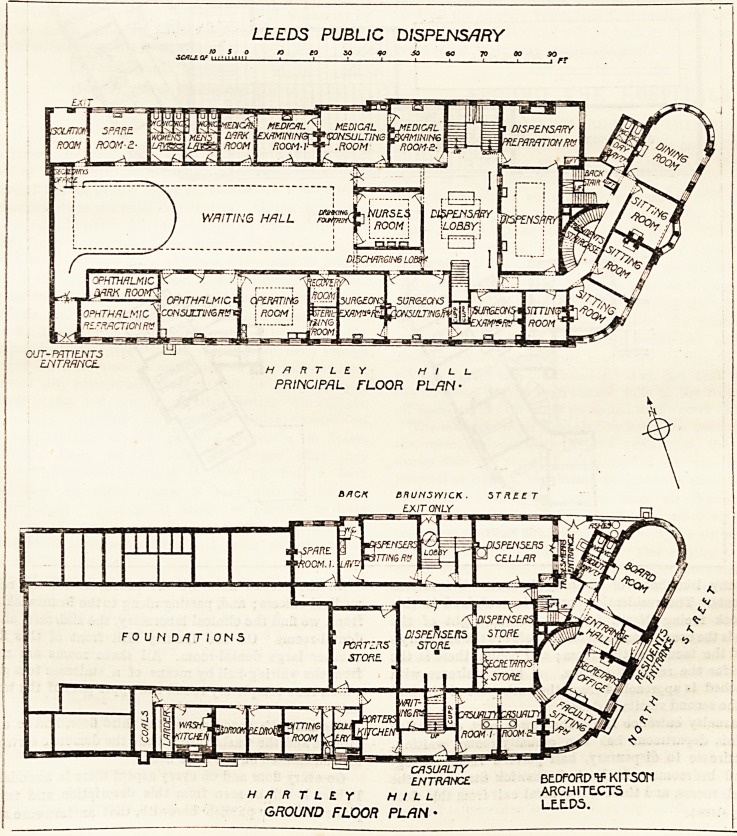


**Figure f2:**